# Highly Efficient Proteolysis Accelerated by Electromagnetic Waves for Peptide Mapping

**DOI:** 10.2174/138920211797248583

**Published:** 2011-09

**Authors:** Qiwen Chen, Ting Liu, Gang Chen

**Affiliations:** School of Pharmacy, Fudan University, 826 Zhangheng Road, Shanghai 201203, China

**Keywords:** Proteomics, Proteolysis, Protease, Electromagnetic waves, Peptide mapping.

## Abstract

Proteomics will contribute greatly to the understanding of gene functions in the post-genomic era. In proteome research, protein digestion is a key procedure prior to mass spectrometry identification. During the past decade, a variety of electromagnetic waves have been employed to accelerate proteolysis. This review focuses on the recent advances and the key strategies of these novel proteolysis approaches for digesting and identifying proteins. The subjects covered include microwave-accelerated protein digestion, infrared-assisted proteolysis, ultraviolet-enhanced protein digestion, laser-assisted proteolysis, and future prospects. It is expected that these novel proteolysis strategies accelerated by various electromagnetic waves will become powerful tools in proteome research and will find wide applications in high throughput protein digestion and identification.

## 1 INTRODUCTION

Proteomics is considered to be the next step in the study of various biological systems after genomics. It is much more complicated than genomics because the protein profiles differ from cell to cell and from time to time while the genome of an organism is more or less constant [1]. Proteomics is the large-scale study of proteins, particularly their functions and structures. As one of the fastest developing areas in biological research, proteomics has drawn more and more research attention [1, 2]. One of its most important tasks is to develop efficient and rapid approaches to identifying various proteins. Peptide mapping is a commonly used strategy in protein identification. Proteins are usually digested into peptides that are subsequently identified by mass spectrometry (MS) to obtain peptide mass fingerprints (PMFs). Protein digestion is a key procedure prior to MS identification. However, conventional in solution tryptic digestion is time-consuming. The typical digestion time is in the range of several hours to half a day [3,4]. Obviously, the extremely long proteolysis time cannot meet the requirements of high-throughput protein identification. It is of high importance to develop novel methods to achieve a highly efficient proteolysis for MS-based peptide mapping.

A variety of approaches have been developed for efficient proteolysis. Recent efforts have been made to fabricate microfluidic enzymatic reactors by immobilizing proteases in the channels of microchips. The bioreactors were further coupled with MS to perform the efficient digestion of low-level proteins and sensitive peptide identification [5-11]. In comparison to the free enzymes in solutions, the enzymes immobilized in microchannels are reported to be much more stable and highly resistant to environmental changes, and provide molecular-level interactions between the immobilized enzymes and the flowing protein substrates. More importantly, the autolysis of protease and the interfering fragments in the digests are minimized. Besides microfluidic chips, proteases have also been immobilized in the inner bores of fused silica capillaries to fabricate flow-through bioreactors for proteolysis [12-14]. In addition, a variety of trypsin-immobilized magnetic particles have been dispersed in protein solutions or packed in microchips and capillaries to carry out efficient proteolysis [15-19].

Electromagnetic wave (i.e. electromagnetic radiation) is a form of energy exhibiting wave-like behavior as it travels through space. Based on the frequency of waves, electromagnetic spectrum consists of radio waves, microwaves, infrared (IR) radiation, visible light, ultraviolet (UV) radiation, X-rays and gamma rays. Among them, microwaves, IR rays, and UV lights have been employed as energy sources to accelerate protein digestion. The digestion time was significantly reduced from 12 to 24 h for the conventional insolution digestion to several minutes or even several seconds for the electromagnetic wave-assisted proteolysis.

This review mainly focuses on the recent advances and the key strategies of efficient proteolysis accelerated by electromagnetic waves. This field of study has grown considerably in the past decade. Reports ranging from microwave-assisted proteolysis to IR-enhanced protein digestion have been published. The following sections will introduce the microwave-accelerated protein digestion, IR-assisted proteolysis, UV-enhanced protein digestion, laser-assisted proteolysis, and future prospects. Various electromagnetic wave-accelerated proteolysis approaches are summarized in Table **1**.

## 2 MICROWAVE-ACCELERATED PROTEIN DIGESTION

Microwaves are electromagnetic waves with wavelengths in the range of one meter to one millimeter. They have found a wide range of applications in the fields of food processing, chemistry, biology, medicine, communication, military, radio astronomy, navigation, spectroscopy, *etc.* Based on the fact that microwaves can heat samples efficiently, they have been widely employed as energy sources to accelerate enzymatic digestion of proteins since the pioneering work of Pramanik and coworkers [20]. During microwave heating, microwaves (usually at a frequency of 2.45 GHz) were allowed to pass through the protein samples in the presence of proteases. Water, proteins, proteases, and other substances in the digestion mixtures absorb energy from the microwaves in a process called dielectric heating. The molecules in the samples are electric dipoles with one end partially positively charged and the other end partially negatively charged. They rotate as they try to align themselves with the alternating electric field of the microwaves. These molecular movements will generate a great deal of heat and enhance the interactions between proteins and proteases so that the digestion efficiency is significantly improved. To date, microwaves have been employed to enhance in-solution, in-gel, in-tip, and on-microsphere protein digestion.

### 2.1 Microwave-Assisted in-Solution Proteolysis

In 2002, Pramanik *et al.* made a breakthrough when they tried to employ microwaves to accelerate conventional in-solution tryptic digestion proteins [20]. They successfully demonstrated the feasibility and performance of this new method for investigating the primary structures of cytochrome *c* (Cyt-*c*), ubiquitin, lysozyme, myoglobin (MYH), and interferon α-2b. The efficient microwave-assisted proteolysis was carried in an inexpensive domestic microwave oven. The proteases they used were trypsin and endoproteases lysine-C. The peptides in the digests were analyzed by matrix-assisted laser desorption ionization time-of-flight MS (MALDI-TOF MS) and liquid chromatography-electrospray ionization MS (LC-ESI MS) techniques. The digestion time was significantly reduced to several minutes in contrast to 12-16 hours for traditional methods. Kinetic investigations indicated that the optimal temperature of the microwave-accelerated proteolysis was 60 °C. About ~70% protein could be digested within 10 min. Most of the expected peptide fragments in the investigated proteins were identified with sequence coverages higher than 80%. No nonspecific cleavage product was found in the digests. It is believed that the use of microwaves in protein identification will be an important advancement in biotechnology and proteome research.

In 2005, Lin and coworkers investigated the effects of solvents on the digestion performances of microwave-assisted proteolysis [21]. It was demonstrated that microwaves accelerated enzymatic digestions of Cyt-*c*, MYH, lysozyme (LYS), and ubiquitin could occur in ammonium bicarbonate aqueous solutions containing acetonitrile, methanol, and chloroform. The results indicated that the digestion efficiency and sequence coverages were significantly increased for the tryptic digestions in the organic solvent-containing solutions that were exposed to microwaves for 10 min. The digestion efficiency was much higher than those obtained under conventional conditions. More importantly, they demonstrated that the investigated solvents could also enhance the digestion efficiency of microwave-assisted in-solution digestion with high sequence coverages. In addition, microwaves could also accelerate the digestion activity of pronase [22,23], α-chymotrypsin [22,23], and pepsin [23] in in-solution proteolysis.

Because microwave-assisted in-solution proteolysis was only demonstrated by digesting several known proteins in the published studies mentioned above, it is of high interest to investigate the digestion performance the microwave-based technique to complex protein samples. In 2006, Gao and coworkers used microwaves to accelerate the tryptic digestion of proteins in human urinary and yeast lysate [24]. The peptide fragments in the digests were further analyzed by capillary LC and ESI MS and MALDI-TOF MS. The method enabled the preparation and digestion of protein mixtures in solution within 6 min. Equivalent digestion efficiency was obtained using microwave-assisted protein enzymatic digestion compared with the standard overnight digestion method. In addition, proteins adsorbed on tiles were also successfully digested by microwave-assisted in solution and the digestion time was reduced to less than 30 min [25]. Under microwave irradiation, digestion completeness depended on digestion temperature, reaction time, enzyme to substrate ratio, and digestion buffer. Reddy *et al.* observed that the number of miss-cleaved peptides and incomplete digestion percentages were often higher than those when the reaction times were longer than a few minutes although all protein molecules in a sample could be digested into peptides within a few minutes under microwave irradiation [26]. Therefore, a reaction time as long as 30 min and approaches to maximize the enzyme activity should be considered if digestion completeness was more important.

### 2.2 Microwave-Accelerated in-Gel Proteolysis

In proteome research, protein mixtures are usually separated using one-dimensional or two-dimensional polyacrylamide gel electrophoresis (PAGE) into bands or spots, which are excised, digested, extracted, and finally identified by MALDI-TOF MS or ESI MS [24]. Two-dimensional electrophoresis combined with MS has significantly offered great promise for the large-scale identification of proteins. However, conventional in-gel digestion is usually performed at 37 °C overnight (12–16 h) and limits the speed of large scale protein identification [27].

In 2005, Juan *et al.* employed microwaves to accelerate in-gel proteolysis [28]. The efficiency of this novel technique was demonstrated by digesting LYS, albumin, conalbumin, and ribonuclease A after they were separated by sodium dodecyl sulphate-polyacrylamide gel electrophoresis (SDS-PAGE). The target protein bands excised from polyacrylamide gel were further treated in trypsin solution and exposed to microwave irradiation. The peptide fragments were further analyzed by MALDI-TOF MS for protein identification. The results indicated that the digestion time was substantially reduced from 16 h for the conventional in-gel digestion to 5 min for microwave-enhanced in-gel digestion with higher sequence coverages. In addition, microwave-accelerated in-gel proteolysis has also been investigated by other groups [20].

### 2.3 Microwave-Accelerated in-Tip Proteolysis

In 2009, Huck and coworkers prepared monolithic stationary phases in pipette tips based on the radical polymerization of a mixture containing glycidylmetha-crylate, divinylbenzene, toluene, and decanol for the covalent immobilization of trypsin [29]. The denatured solution of MYH, bovine serum albumin (BSA) and α-casein were aspirated into the prepared in-tip proteolysis bioreactors in a microwave oven for 2 min at 70 W. An automated robotic system was employed to carry out fast and reproducible sample treatment. The molecular weights of the peptides in the obtained digests were determined by MALDI-TOF MS and LC-ESI MS. The results indicated that the three proteins were well digested with high sequence coverages of 89%, 78%, and 83% for MYH, BSA and α-casein, respectively. Furthermore, microwave-assisted in-tip proteolysis ensured very fast and reproducible results, even at a protein concentration as low as 10 μg/mL.

### 2.4 Microwave-Accelerated Proteolysis Enhanced by Microwave Absorbers

It was supposed that the time required for microwave-assisted enzymatic digestion might be further reduced if appropriate microwave absorbers were added to the digestion solution. Recently, Hasan *et al.* used titania nanoparticles to enhance the digestion efficiency of microwave-assisted in-solution proteolysis [30]. The bare titania nanoparticles served as multifunctional nanoprobes (desalting, accelerating, and affinity probes) for the efficient enrichment of phosphopeptides from microwave-assisted tryptic digestion of phosphoproteins (α-casein, β-casein and milk). The peptides in the digests were subsequently identified by ESI MS and MALDI MS. The developed approach significantly reduced the digestion time to 45 s instead of 12–16 h required for traditional in-solution proteolysis. The dispersed titania nanoparticles could adsorb microwave radiation that accelerated the activation of trypsin for efficient digestion of phosphoproteins and the ionization of phosphopeptides were enhanced. Higher sensitivity and sequence coverage of phosphopeptide were obtained because the digested and partially digested phosphoproteins were concentrated onto the surface of titania nanoparticles due to the high binding affinity and selectivity of titania nanoparticles toward phosphopeptides and phosphoproteins.

In 2007, Chen *et al.* dispersed negatively charged magnetic beads in the tryptic digestion solution of Cyt-*c* and MYH in a microwave oven [16]. Both proteins were well digested within 1 min with satisfactory sequence coverages.

### 2.5 Microwave-Accelerated Proteolysis on Magnetic Particles

Because magnetic particles were reported as excellent absorbers of microwave radiations, trypsin was immobilized on them to perform microwave-assisted proteolysis [16,17, 31-33]. The magnetic nanoparticles served as not only substrate for enzyme immobilization, but also excellent microwave absorbers. The time required for microwave-assisted enzymatic digestion were further reduced because the capacity to absorb microwave radiation led to rapid heating of the magnetic particles. After digestion, the magnetic particles could be easily separated with the aid of a small magnet. In 2007, Chen and coworker immobilized trypsin covalently on the surface of magnetic particles *via* amimo group-containing silane coupling agent for the efficient proteolysis of MYH [16]. The digestion time was significantly reduced to 1 min. In addition, Deng *et al.* prepared a series of trypsin-immobilized magnetic nanoparticles for efficient proteolysis in a microwave oven [17, 31-33]. The digestion efficiency was demonstrated in combination with the MS-based peptide mapping of BSA, MYH, Cyt-*c*, and some real samples. The digestion process was very simple due to the easy manipulation of magnetic nanoparticles. Complete protein digestion could accomplish in the range of 15 to 45 s, without the need for any complicated reduction and alkylation procedures. The digestion efficiency and sequence coverage were equivalent to or better than 12-h conventional in-solution digestion.

## 3 IR-ASSISTED PROTEOLYSIS

As an important form of electromagnetic wave, the IR region of the electromagnetic spectrum lies between visible radiation and microwaves. IR radiation has wavelengths ranging from 760 nm to 1 mm and has found a wide range of applications in domestic and industrial heating, communications, military, tracking in military, spectroscopic analysis, astronomy, medical and health care [34,35]. High-efficiency heating was achieved by matching the wavelengths of the IR heaters to the absorption characteristics of the materials [34,36]. It is of high interest to demonstrate the possibility of employing IR ray as an energy source to enhance the efficiency of the conventional in-solution proteolysis.

### 3.1 IR-Assisted in-Solution Proteolysis

IR radiation has been employed to accelerate tryptic proteolysis for peptide mapping [37]. Fig. (**1**) illustrates the schematic of the IR-assisted proteolysis system. It consists of an IR lamp, a case fan, a temperature controller connected with a thermocouple, and an iron case. Both the IR lamp and the thermocouple were assembled in the iron case. The case fan was fixed on the sidewall of the case to drive cool air inside to adjust the temperature. The iron case has a door and several heat elimination holes. The temperature controller could turn on or turn off the case fan when the temperate in the case was higher or lower than 37 °C, respectively.

Protein solutions containing trypsin in sealed transparent Eppendorf tubes were allowed to digest under an IR lamp. The feasibility and performance of the novel proteolysis approach were demonstrated by the digestion of BSA and MYO and the digestion time was significantly reduced to 5 min. The obtained digests were identified by MALDI-TOF MS with the sequence coverages of 69% (BSA) and 90% (MYO) that were much better than those obtained by conventional in-solution tryptic digestion.

α-Chymotrypsin is another commonly used protease that selectively catalyzes the hydrolysis of peptide bonds on the C-terminal side of tyrosine, phenylalanine, tryptophan, and leucine. The typical time of in-solution chymotryptic proteolysis is in the range of 12 to 24 h [38,39]. In 2008, Wang *et al.* employed IR radiation to enhance the efficiency of in-solution chymotryptic proteolysis [40]. Protein solutions containing chymotrypsin were allowed to digest under an IR lamp at 37 °C for 5 min. Fig. (**2**) shows the PMFs of the chymotryptic digests of 200 ng/μL BSA and 200 ng/μL Cyt-*c* obtained by using 5-min IR-assisted digestion. Both samples were well digested and positively identified. It was found that 29 and 13 chymotryptic peptides were matched with the corresponding amino-acid sequence coverage of 41% and 75% for BSA and Cyt-*c*, respectively. The results indicate that 254 out of the 607 possible amino acids of BSA 130 and 79 out of the 104 possible amino acids of Cyt-*c* have been identified. The results were comparable to those obtained by using conventional in-solution digestion. The suitability of IR-assisted chymotryptic proteolysis to complex proteins was demonstrated by digesting human serum.

### 3.2 IR-Assisted in-Gel Proteolysis

Recently, IR radiation was also employed to enhance the efficiency of in-gel proteolysis for MS-based protein identification [41]. After SDS-PAGE, the target protein bands excised from polyacrylamide gel were cut into small pieces that were further treated in trypsin solution. Subsequently, the wet gel pieces sealed in transparent Eppendorf tubes were exposed to an IR lamp to perform IR-assisted in-gel digestion. To demonstrate the performance of the novel digestion approach, it was employed to digest BSA and Cyt-*c* in polyacrylamide gels after SDS-PAGE separations. The results indicated that IR radiation substantially enhanced the efficiency of in-gel proteolysis and the digestion time was significantly reduced to 5 min compared to 16 h for conventional in-gel digestion. The obtained digests were further identified by MALDI-TOF MS with improved sequence coverages.

Figs. (**3A** and **3B**) show PMFs of the extracted digests of 1 µg BSA (500 ng/µL ( 2 µL) and 1 µg Cyt-*c* (500 ng/µL ( 2 µL) from gel pieces obtained by using 5-min IR-assisted in-gel digestion. Both samples were well digested and positively identified. It was found that 37 and 12 tryptic peptides were matched with the corresponding amino-acid sequence coverage of 48% and 67% for BSA and Cyt-*c*, respectively. The suitability of IR-assisted in-gel proteolysis to real protein samples was demonstrated by digesting and identifying human serum albumin in gel separated from human serum by SDS-PAGE.

### 3.3 IR-Assisted on-Plate Proteolysis

**Fig. (4):**
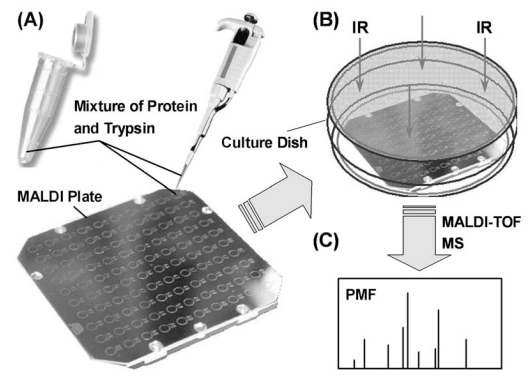
Schematic showing the process of IR-assisted on-plate proteolysis. (**A**) Dropping the mixture of protein and trypsin on MALDI palte; (**B**) enclosing the plate in a glass culture dish and exposing to IR radiation to accelerate the digestion; (**C**) MALDI-TOF-MS peptide mapping. Reprinted with permission from ref. [42].

MALDI-TOF MS has been widely used in protein chemistry and proteomics research for the identification of proteins. Protein samples were usually digested into peptides with enzymes. Subsequently, the obtained digests were dropped on MALDI plates to perform MS measurements. To simplify the analysis process, on-plate proteolysis approaches were developed by combining digestion and spotting into one procedure. IR radiation has been employed to enhance the digestion efficiency of in-solution tryptic proteolysis on plates [42]. Protein solutions containing trypsin was dropped on the spots of a MALDI plate that was exposed to an IR lamp for 5 min to perform high efficient proteolysis (Fig. **4**). A layer of moist circular filter paper needed to be sandwiched between the plate and the bottom of the culture dish to humidify the enclosed cavity. The novel IR-assisted on-plate proteolysis approach has been coupled with MALDI-TOF MS for the digestion and peptide mapping of BSA and Cyt-*c.*

### 3.4 IR-Assisted in-Chip Proteolysis

In addition, IR radiation was also employed to accelerate on-chip proteolysis [43]. Liu *et al.* developed an inflation bulb-driven microfluidic reactor for IR-accelerated proteolysis. This novel proteolysis system mainly consisted of an inflation bulb driving system, a simple cross poly(methyl methacrylate) microchip, and a temperature-controllable IR radiation system. The gas pressure generated from an inflation bulb was employed to drive protein and trypsin solutions to flow into the main channel of the microchip *via* two capillaries and the injection channel. When the two solutions were mixed in the channel, the protein was rapidly digested by trypsin under IR radiations. The peptides in the digests accumulated in the product reservoir of the microchip were subsequently identified by MS. Fig. (**5**) illustrates the PMF spectra of the tryptic digests of 100 ng/µL hemoglobin (HEM) and 100 ng/µL LYS obtained by using IR-assisted on-chip proteolysis. Both protein samples were digested and positively identified. The identified peptides in the digests of HEM and LYS were presented in the insets of Figs. (**5A** and **5B**), respectively. It was found that a total of 16 and 8 tryptic peptides were matched with the amino-acid sequence coverages of 77% and 42% for HEM and LYS, respectively. The results indicated that IR radiation could significantly enhance the on-chip proteolysis and the digestion time was substantially reduced to 5 min. The present proteolysis setup is simple and efficient and will find wide applications in high throughput protein digestion.

### 3.5 IR-Assisted on-Microsphere Proteolysis

More recently, Bao *et al.* developed a novel proteolysis approach based on IR radiation and trypsin-immobilized silica microspheres [44]. As illustrated in Fig. (**6**), trypsin was covalently immobilized on the surface of silica microspheres *via *3-glycidoxypropyltrimethoxysilane. Protein solutions containing the prepared trypsin-immobilized microspheres in sealed transparent Eppendorf tubes were allowed to digest under an IR lamp at 37 °C for 5 min. The on-microsphere proteolysis approach was employed to digest BSA and Cyt-*c.* The obtained digests were identified by MALDI-TOF MS with the sequence coverages of 54% (BSA) and 83% (Cyt-*c*) that were better than those obtained by conventional in-solution tryptic digestion. The suitability of the digestion approach to complex proteins was demonstrated by digesting human serum.

### 3.6 Possible Mechanism of IR-Assisted Proteolysis

The significantly enhanced digestion efficiency of the present IR-assisted proteolysis approach can be attributed to the IR radiation. Photons in the IR region of the electromagnetic spectrum have much less energy than photons in the visible or UV regions. They could only excite the vibrations in molecules (such as trypsin and proteins) in the modes of stretching, bending, rocking, and twisting [37]. These vibrations increased the frequency of the interaction between trypsin and the protein molecules, resulting in highly efficient proteolysis. The IR-induced vibrations of proteins might also lead to more cleavage sites of proteins exposed to trypsin, resulting in easier cleavage of peptide chains. It might be the reason why there were more matched peptides in the PMFs of the digests obtained by using IR-assisted digestion.

## 4 UV-ENHANCED ON-PLATE PROTEIN DIGESTION

UV light is a kind of electromagnetic radiation with a wavelength ranging from 10 nm to 400 nm. It was also employed to accelerate tryptic digestion of 200 ng/µL BSA BSA and 200 ng/µL Cyt-*c* on MALDI plate for MALDI-TOF MS peptide mapping [42]. The wavelength of the UV light was 365 nm while the digestion time was 5 min. A total of 18 and 6 tryptic peptides from BSA and Cyt-*c* were found matched with the corresponding sequence coverage of 29% and 52% for BSA and Cyt-*c*, respectively. In comparison with the results of 5-min on-plate digestion in a dark enclosure, the sequence coverages of BSA and Cyt-*c* increased from 12% to 29% and from 42% to 52%, respectively. The results indicated that UV radiation could also enhance the efficiency of on-plate tryptic proteolysis to some extent. However, the results of IR-assisted on-plate digestion mentioned above were much better than those of UV-assisted on-plate digestion, implying IR radiation showed higher acceleration performance towards proteolysis than higher energy photons in the UV range. In addition, UV radiation was much more dangerous than IR radiation and was not easy to handle.

## 5 LASER-ASSISTED PROTEOLYSIS

**Fig. (7):**
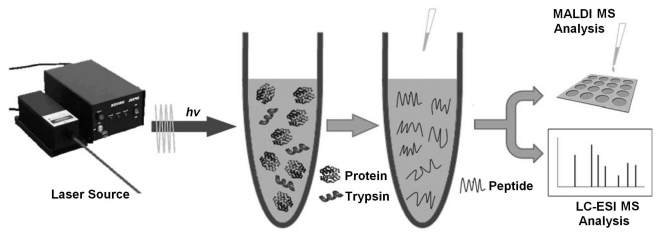
Schematic illustration of laser-assisted in-solution proteolysis. Reprinted with permission from ref. [45].

A laser beam is a stream of focused coherent light in a single wavelength. There are many different kinds of lasers that have found a wide range of technological applications. Recently, Deng and co-workers used near IR laser (808 nm) as an energy source to promote in-solution, in-gel, and on-plate tryptic proteolysis (Fig. **7**) [45]. The digestion time was significantly reduced to less than 1 min. The results indicate that laser-assisted proteolysis coupled with MALDI-TOF MS is a promising strategy for efficient protein digestion and peptide mapping. It was also demonstrated that this laser-assisted digestion protocol was also applicable to the efficient digestion of proteins at a low concentration of 25 ng/µL. In addition, the novel proteolysis approach was successfully applied in the rapid digestion of the proteins in rat-brain extracts, indicating the strong potential of this straightforward, fast, efficient, and low-cost approach in high-throughput proteome analysis.

## 6 SUMMARY

It can be concluded that electromagnetic wave-assisted proteolysis coupled with various MS techniques is a promising strategy for the efficient protein digestion and peptide mapping. In the past decade, a series of electromagnetic waves, including microwaves, UV radiations, IR rays, and laser radiations, have been successfully employed to accelerate protein digestion in solution and gels. With the assistance of these electromagnetic waves, digestion time was substantially reduced to several seconds to several minutes compared to 12 to 24 hours for conventional in-solution digestion. The digests were identified by MS with sequence coverages that were comparable to or even better than those obtained by using conventional in-solution proteolysis. The suitability of electromagnetic wave-assisted proteolysis to complex proteins has been demonstrated by digesting some real biological samples. In addition, these electromagnetic radiations can be easily integrated into LC, capillary electrophoresis, MS, and other automation analysis systems for high-throughput protein identification. Among these efficient digestion strategies, the IR-assisted digestion approach indicates greater promise for rapid and high throughput protein identification because IR radiation is safer than other electromagnetic waves. Undoubtedly, the ease, simplicity, efficiency, and low cost of these novel proteolysis approaches indicate they may find further application in automated analysis of large sets of proteins. Future research attentions of electromagnetic radiation-assisted proteolysis will focus on their possible mechanisms, kinetics, integration with automation analysis systems, and their applications in the identification of complex proteins in real samples.

## Figures and Tables

**Fig. (1):**
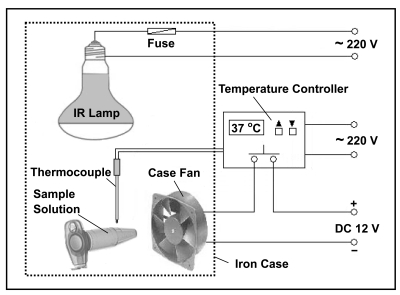
Schematic of a IR-assisted proteolysis system. Reprinted with permission from ref. [37].

**Fig. (2):**
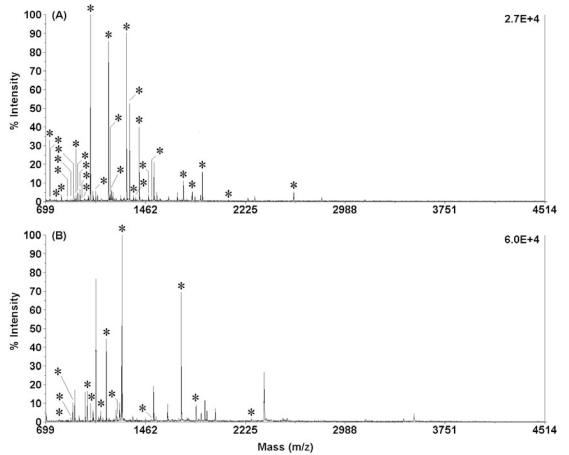
MALDI-TOF mass spectra of the digests of 200 ng/µL BSA (**A**) and 200 ng/µL Cyt-*c* (**B**) in 10 mM NH_4_HCO_3_ buffer solution (pH 8.1) obtained by using 5-min IR-assisted digestion (chymotrypsin/substrate ratio, 1:40; digestion temperature, 37 °C; all matched peptides were marked with “*”). Reprinted with permission from ref. [40].

**Fig. (3):**
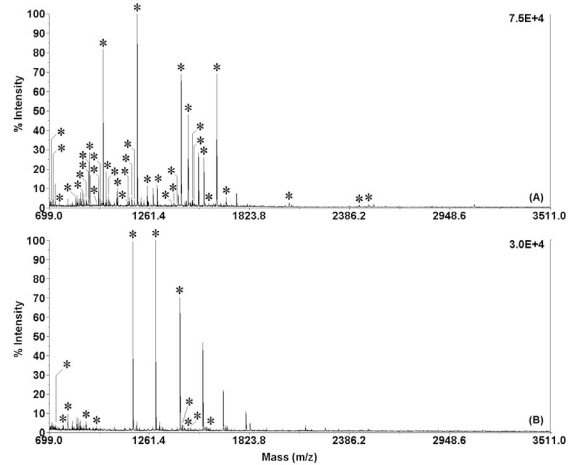
MALDI-TOF mass spectra of the extracted digests of 1 µg BSA (500 ng/µL × 2 µL, **A**) and 1 µg Cyt-*c* (500 ng/µL × 2 µL, **B**) from gel pieces obtained by using 5-min IR-assisted in-gel digestion at 37 °C. All matched peptides were marked with “*”. Reprinted with permission from ref. [41].

**Fig. (5):**
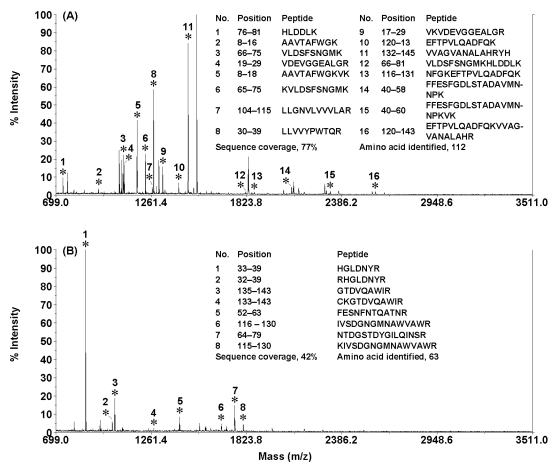
MALDI-TOF mass spectra of the digests of 100 ng/µL HEM (**A**) and 100 ng/µL LYS (**B**) in 20 mmol/L NH_4_HCO_3_ buffer solution (pH 8.0) obtained by using IR-assisted on-chip proteolysis (trypsin/substrate ratio, ~1:40; digestion time, 5 min; driving pressure, 0.7 kg/cm^2^). Matched peptides were marked with “*”. Reprinted with permission from ref. [43].

**Fig. (6):**
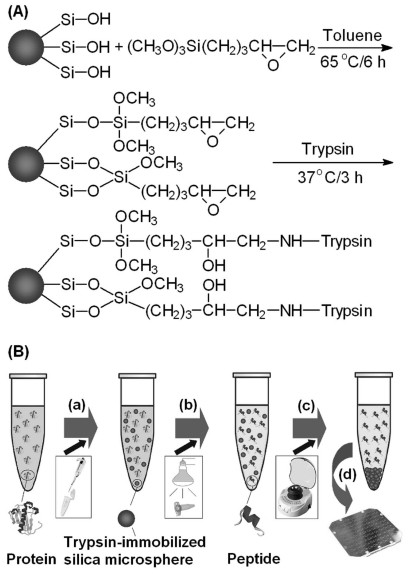
Schematic diagrams showing (**A**) the immobilization process of trypsin on the surface of a silica microsphere and (**B**) the procedure of IR-assisted proteolysis using trypsin-immobilized silica microspheres. (a) Dispersing trypsin-immobilized silica microspheres in protein solution; (b) digesting under an IR lamp; (c) isolating trypsin-immobilized silica microspheres by centrifugation, (d) MALDI-TOF-MS peptide mapping. Reprinted with permission from ref. [44].

**Table 1:** Electromagnetic Wave-Assisted Proteolysis Approaches

Proteolysis approaches	Electromagnetic wave	Digestion medium	Protease	Digestion time	Ref.
In-solution proteolysis	Microwave	Water	Trypsin	10 min	[20]
In-solution proteolysis	Microwave	Water	Endoproteases lysine-C	10 min	[20]
In-solution proteolysis	Microwave	Organic solvent/water	Trypsin	10 min	[21]
In-solution proteolysis	Microwave	Water	Pronase	10 min	[22]
In-solution proteolysis	Microwave	Water	Pronase	3 min	[23]
In-solution proteolysis	Microwave	Water	α-Chymotrypsin	10 min	[22]
In-solution proteolysis	Microwave	Water	α-Chymotrypsin	3 min	[23]
In-solution proteolysis	Microwave	Water	Pepsin	3 min	[23]
In-solution proteolysis	Microwave	Water	Trypsin	1 min	[24]
In-gel proteolysis	Microwave	Gel/water	Trypsin	5 min	[24]
In-solution proteolysis	Microwave	Tiles/Water	Trypsin	30 min	[25]
In-solution proteolysis	Microwave	Water	Trypsin	30 min	[26]
In-gel proteolysis	Microwave	Gel/water	Trypsin	5 min	[28]
In-gel proteolysis	Microwave	Gel/water	Trypsin	15 min	[20]
In-tip proteolysis	Microwave	Monolith/water	Immobilized trypsin	2 min	[29]
In-solution proteolysis	Microwave	Titania particles/water	Trypsin	45 s	[30]
In-solution proteolysis	Microwave	Magnetic particles/water	Trypsin	1 min	[16]
Proteolysis on magnetic particles	Microwave	Magnetic particles/water	Immobilized trypsin	15 s	[17]
Proteolysis on magnetic particles	Microwave	Magnetic particles/water	Immobilized trypsin	15 s	[31]
Proteolysis on magnetic particles	Microwave	Magnetic particles/water	Immobilized trypsin	15 s	[32]
Proteolysis on magnetic particles	Microwave	Magnetic particles/water	Immobilized trypsin	45 s	[33]
In-solution proteolysis	IR radiation	Water	Trypsin	5 min	[37]
In-solution proteolysis	IR radiation	Water	α-Chymotrypsin	5 min	[40]
In-gel proteolysis	IR radiation	Gel/water	Trypsin	5 min	[41]
On-plate proteolysis	IR radiation	Water	Trypsin	5 min	[42]
In-chip proteolysis	IR radiation	Water	Trypsin	6 min	[43]
On-microsphere proteolysis	IR radiation	Water	Immobilized trypsin	5 min	[44]
On-plate proteolysis	UV radiation	Water	Trypsin	5 min	[42]
In-chip proteolysis	Laser radiation	Water	Trypsin	30 s	[45]
In-gel proteolysis	Laser radiation	Gel/water	Trypsin	45 s	[45]
On-plate proteolysis	Laser radiation	Water	Trypsin	5 s	[45]

## ABBREVIATIONS

MS: = Mass spectrometry
TOF: = Time-of-flight
MALDI: = Matrix-assisted laser desorption/ionization
ESI: = Electrospray ionization
IR: = Infrared
UV: = Ultraviolet
BSA: = Bovine serum albumin
Cyt-*c*: = Cytochrome *c*
MYH: = Myoglobin
HEM: = Hemoglobin
LYS: = Lysozyme
LC: = Liquid chromatography
PAGE: = Polyacrylamide gel electrophoresis
SDS: = Sodium dodecyl sulphate
PMF: = Peptide mass fingerprinting
